# Reciprocal changes of serum adispin and visfatin levels in patients with type 2 diabetes after an overnight fast

**DOI:** 10.1590/2359-3997000000147

**Published:** 2016-01-01

**Authors:** Ioannis Legakis, Timos Mantzouridis, George Bouboulis, George P. Chrousos

**Affiliations:** 1 Iaso General Hospital Athens Greece Iaso General Hospital, Athens, Greece; 2 Euromedica High Technology S.A Athens Greece Euromedica High Technology S.A. Athens, Greece; 3 Aretaieion University Hospital Athens Medical School Athens Greece Aretaieion University Hospital, Athens Medical School, Athens, Greece; 4 First Department of Pediatrics University of Athens School of Medicine Athens Greece First Department of Pediatrics at the University of Athens School of Medicine, Athens, Greece

**Keywords:** Adipsin, visfatin, type 2 diabetes, fasting, humans

## Abstract

**Objective:**

In order to elucidate the interrelationship of adipokines in glucose hemiostasis, we determined the concentration of visfatin and adipsin in blood samples in patients with type 2 diabetes and age-matched controls after an overnight fast.

**Subjects and methods:**

We enrolled 37 patients with known type 2 diabetes -21 males and 16 females, aged 62.95 ± 15.72 years and 43 controls- 28 males and 15 females, aged 60.79 ± 12.67 years. Blood samples were collected after an overnight fast and routine biochemical parameters such as glucose, cholesterol, HDL, LDL, triglycerides along with Hb1Ac, insulin and c-peptide, in addition to circulating visfatin and adipsin were determined in all samples. Data were considered significant at a level of p < 0.05.

**Results:**

In patients with type 2 diabetes, circulating adipsin levels were decreased and inversely related with glucose levels while circulating visfatin was increased significantly in the fasting state.

**Conclusion:**

These results implicate the adipokines adipsin and visfatin as possible participants in the pathogenesis of type 2 diabetes.

## INTRODUCTION

Adult onset diabetes or type 2 diabetes is best described by the presence of insulin resistance coexisting with impaired insulin secretion. Both *in vitro* and *in vivo* studies associate diverse fat topography and adipocyte metabolism as causative factors in the pathogenesis of the disease (1). Most recently, experimental and clinical studies suggest that novel adipokines such as adipsin and visfatin might have an energetic role in the cascade of the events leading to diabetes mellitus. Namely, adipsin has a beneficial role in maintaining β cell function and visfatin plays an important role in glucose metabolism (2,3).

In order to elucidate the interrelationship of these adipokines in glucose homeostasis *in vivo*, we determined the concentration of visfatin and adipsin in blood samples in patients with type 2 diabetes and age-matched controls after an overnight fast.

## SUBJECTS AND METHODS

We enrolled 37 patients with known type 2 diabetes -21 males and 16 females (mean age 62.95 ± 15.72 years; range: 47-7) and 43 controls- 28 males and 15 females (mean age 60.79 ± 12.67 years; range: 48-73). Type 2 diabetes was medically confirmed by using a 75-g oral glucose tolerance test. Those patients were eligible who had a history of at least 3 years of type II diabetes, without any complications of diabetes, and who were treated only by oral glucose-lowering medications. Emphasis was given to the similarity between control and T2DM patient groups in terms of BMI, age and gender. Blood samples were collected after an overnight fast and routine biochemical parameters such as glucose, cholesterol, HDL-C, LDL-C, triglycerides along with HbA1c, insulin and c-peptide were also determined in all samples. BMI was calculated as weight in kilograms divided by the square of height in meters. Exclusion criteria for this study were 1) BMI ≥ 40 kg/m^2^ and 2) concurrence of any systemic disease or medication use. Written informed consent was obtained from all study participants. Serum visfatin was measured using visfatin C-Terminal (Human) enzyme immunoassay kit with a sensitivity of 0.1 ng/mL by Phoenix Pharmakeuticals, Inc. Serum adipsin was measured using human adipsin/factor D ELISA kit with a minimum detectable dose of 4 pg/mL made by RayBiotech, Inc. The procedures provided with these kits were applied strictly as mentioned. Insulin resistance was calculated by the Homeostasis Model of Assessment of Insulin Resistance (HOMA-IR).

All data are presented as mean values ± standard deviation of the mean (SD). Analysis of variance and T-test of independent means were used for statistical analysis. Data were considered significant at a level of p < 0.05.

## RESULTS

As presented in Table 1, the mean values of anthropometric measures were not significantly different between the diabetic group and controls.

**Table 1:** Comparison of anthropometric and metabolic measurements in diabetic patients and controls

Variables	Diabetics Mean ± SD	Normals Mean ± SD	P
Age (years)	62.9 ± 22.37	60.79 ± 18.9	0.63
BMI (kg/m^2^)	25.03 ± 1.19	25.01 ± 1.24	0.92
SBP (mmHg) DBP (mmHg)	115.2 ± 7.364 74.08 ± 6.54	114.6 ± 7.031 73.44 ± 6.41	0.716 0.66
Total cholesterol (mg/dL) HbA1c (%)	189.9 ± 46.36 6.6 ± 0.46	211 ± 44.62 5.0 ± 0.54	0.033 < 0.0001
HDL-C (mg/dL)	33.59 ± 9.17	36.67 ± 9.96	0.15
LDL-C (mg/dL)	112.9 ± 43.64	138.4 ± 39.46	0.0076
Triglycerides (mg/dL)	246 ± 288.0	184 ± 103.2	0.19
Total lipids (mg/dL)	668.9 ± 322.8	644.8 ± 149.1	0.662
Fasting insulin (μU/mL)	29.15 ± 21.38	11.99 ± 4.765	< 0.0001
Fasting C-peptide (ng/mL)	0.997 ± 0.780	1.145 ± 0.912	0.442
HOMA-IR	4,331 ± 3.197	1.568 ± 0.625	< 0.0001
Adipsin (pg/mL)	74.30 ± 12.51	117.1 ± 5.304	< 0.0001
Visfatin (ng/mL)	4.968 ± 2.138	2.891 ± 0.6168	< 0.0001

According to our results, circulating adipsin was decreased in diabetes compare to normal controls (Adipsin diabetics: 74.30 ± 12.51 pg/mL *versus* normals: 117.1 ± 5.03 pg/mL, p < 0.0001). Moreover adipsin was inversely correlated with fasting glucose levels (r = -0.619, p < 0.00006). In diabetics, positive correlation was detected between fasting glucose levels, triglycerides (r = 0.610, p < 0.00006) and total lipids (r = 0.506, p < 0.001). As expected cholesterol levels showed positive correlation with LDL cholesterol (r = 0.887, p < 0.001) and total lipids (r = 0.540, p < 0.0006) in the diabetic group. On the other hand, circulating visfatin was increased significantly in diabetics compare to normal controls (Visfatin – diabetics: 4.968 ± 2.13 ng/mL *versus* normals: 2.891 ± 0.61 ng/mL, p < 0.0001). No other correlation between visphatin and measured metabolic parameter was detected.

## DISCUSSION

According to early observations in the late 1980s, adipocytes are responsible for a number of factors that are subjected to metabolic changes. In nowdays, we know that the adipose tissue serves not only as an energy storage organ but also as an endocrine organ that secretes a variety of hormones, so called-adipokines, into the circulation with distinct target receptors in many tissues (i.e.: hypothalamus, muscle and liver). Based on recent scientific literature, the adipose-derived hormones influence energy metabolism, with its cells, adipocytes, exhibiting both paracrine and endocrine activity (1).

Several of these adipocyte hormones are well recognized and described concerning their structure and biological role. They play an essential role in the preservation of energy homeostasis by regulating insulin secretion, glucose and lipid metabolism, and in some cases directly affecting insulin action in skeletal muscle, liver, and adipose tissue. In particular, adipsin was the first major protein secreted from white fat to be identified, after lipoprotein lipase. It was initially detected to increase with differentiation of adipocytes (4). Althought, early observations support the view that adipsin could function as lipostatic signal based on the decreased expression of the adipsin gene and circulating protein levels in animal models of obesity, that was not supported in clinical data (5). Interestingly, it has been shown that adipsin has a beneficial role in maintaining β cell function. In particular, animal studies confirmed that genetically lacking adipsin coexists with glucose intolerance due to insulinopenia. Moreover, replenishment of adipsin to diabetic animals treated hyperglycemia by boosting insulin secretion (3). Additionally, a newly discovered adipokine, visfatin, which is produced and secreted primarily by visceral white fat, has been shown to exert insulinmimetic effects both *in vitro* and *in vivo* through its actions to insulin receptor (6,7). Visfatin stimulated glucose uptake in differentiated skeletal muscle cells by increasing glucose transporter type 4 (GLUT4) mRNA and protein levels. According to clinical studies, visfatin serum levels are associated with type 2 *diabetes mellitus* independent of insulin resistance and obesity (6). Concerning the controversies regarding the association of visfatin with overweight/obesity, type 2 diabetes mellitus, insulin resistance (IR), metabolic syndrome and cardiovascular disease in published articles, a meta-analysis was performed suggesting that the use of visfatin may be a promising predictive tool for these disorders (7). Recent clinical data showed that in type 2 diabetics, although visceral adiposity index (VAI) exhibit a significant positive correlation with visfatin serum levels, no significant differences was detected for adipsin, even when a two-step cluster analysis was performed in order to indentify altered adipocytokine profiles (8).

In our study, the reciprocal changes of adipsin and visfatin levels in patients with type 2 diabetes in the fasting state implicate these hormones with both paracrine and endocrine functions as possible participants in the in the pathogenesis of type 2 diabetes. In type II diabetes, insulin resistance and obesity together cause mild to moderate hypertriglyceridemia and also cause reduction of HDL-C. Excessive production of TG-rich lipoproteins and its low clearance by lipoprotein lipase in diabetic patients may lead to hypertriglyceridemia (9). The aforementioned metabolic pattern was also confirmed in our study.

In conclusion, the distinct metabolic profile of the adipokines adipsin and visfatin in patients with type 2 diabetes enhance the potential role of the adipose tissue in the modulation of energy balance and glucose homeostasis have a potential clinical relevance as biomarkers for insulin sensitivity.
